# Development of Priority Assistive Product Lists in Three African Countries

**DOI:** 10.3390/ijerph21111393

**Published:** 2024-10-23

**Authors:** Emma M. Smith, Ying Zhang, Laura Ligthart, Ikenna D. Ebuenyi

**Affiliations:** 1 Assisting Living and Learning Institute, Maynooth University, W23 H2H6 Maynooth, Ireland; 2 Faculty of Science, Vrije Universiteit, 1081 HV Amsterdam, The Netherlandsligthartlaura@gmail.com (L.L.); 3 Department of Rehabilitation Science and Technology, University of Pittsburgh, Pittsburgh, PA 15260, USA; ikenna.ebuenyi@pitt.edu

**Keywords:** assistive technology, products, policy, African

## Abstract

National Assistive Product Lists (APLs) play an important role in improving access to assistive products (APs). Assistive products are critical to enhancing the health, well-being, and quality of life of persons with disabilities and other functional limitations, including those associated with aging. Comparing national APL development across Malawi, Liberia, and Sierra Leone may provide insight into the differences between the resulting national APLs and how to enhance AP service delivery systems. The aim of this study was to compare how the World Health Organization’s 5Ps model (people, personnel, policy, provision, products) influenced national APL development across Malawi, Liberia, and Sierra Leone. To achieve this aim, we conducted a series of qualitative interviews with representatives of key government and non-state organizations (n = 12) who had been involved in the development of the APL in each of the three countries. We used directed content analysis to review and analyze the resulting data, with the 5Ps representing the 5 areas of analysis. Our results found substantial differences between the APLs of the three countries, which were substantially influenced by the needs of assistive technology users in each of the respective countries (people). This was evident in the fact that product selection criteria differed across countries, with the most critical factor being population need. Provision systems were generally fragmented and depended heavily on donors, with a lack of coordination between the public and private sectors. None of the countries had or produced a standalone AT policy in the APL development process. County-specific factors also influenced the APL differences between countries. Our research concludes that national APLs will vary substantially if they are developed collaboratively, considering the needs of the population with consideration for the country’s context and existing policies and systems.

## 1. Introduction

Assistive products (APs) are any external products that promote independence and functioning for individuals [1]. Appropriate APs are critical to enhancing the health, well-being, and quality of life of persons with disabilities and other functional limitations, including those associated with aging, while helping to reduce the costs of healthcare [1]. The Global Report on Assistive Technology, co-produced by the World Health Organization (WHO) and The United Nations Children’s Fund (UNICEF), estimates that over 2.5 billion individuals currently need AP, and this number is expected to rise above 3.5 billion by 2050 due to the aging population and increasing prevalence of non-communicable diseases [2]. However, despite the increasing number of people in need of APs, only one in ten of the needs is being met [1].

Access to AP varies by country, with some countries reaching coverage of up to 90% (i.e., 90% of those who require AP have access to it) and others as low as 3% coverage [2]. Data suggest access to AP is generally lower in lower- and middle-income countries (LMICs) [2]. For example, one study suggests only 5% of people with amputation in LMICs have access to AP [3]. Estimates suggest that the total population in Africa is more than 1 billion, and over 200 million people among them need at least one AP [4]. However, only about 15–25% of people with disabilities or functional limitations in this context have access to AP [5]. In the World Report on Disability, the WHO estimated that approximately 15.6% of people living in Africa are persons with disabilities (PWD) [6]. Financial constraints, a lack of awareness and skilled service providers, limited availability, weak governance regarding the rights of people with disabilities, and other factors all impede the provision of assistive technology (AT) [1]. A lack of affordable and appropriate access to AP in many LMICs results in high out-of-pocket costs [1,7].

The UN Convention on the Rights of Persons with Disabilities affirms access to assistive products as critical to achieving progressive realization of the rights enshrined within [8]. The provision of AT is critical to realizing the recommendations of the CRPD and UN Sustainable Development Goals (SDGs) [9,10]. Therefore, WHO developed a priority assistive products list (APL) to improve access to affordable and high-quality AP [1,11]. This list includes 50 APs selected based on need, cost-effectiveness, and impact on the lives of beneficiaries. This list is not restrictive and is intended to guide member states to develop national APLs based on their contexts, needs, and available resources to enhance access to AP [1]. To facilitate AT provision, the World Health Assembly (WHA) resolution 71.8 calls on member states to develop AT policies and systems (WHA, 2018). Public health policies that promote access to assistive products through universal healthcare are central to realizing the rights of persons with disabilities, including the right to access health and participation more broadly.

MacLachlan and Scherer (2018) suggested adopting a systemic approach to APL development. This means that the APL development encompasses not only the APL implementation but also the development of the overall AT system [12]. AT systems are defined as ‘the development and application of organized knowledge, skills, procedures, and policies relevant to the provision, use, and assessment of assistive products’ [13]. To address the need for systemic approaches to policy development, the WHO developed a theoretical model known as the 5P model (people, policy, products, provision, personnel) to assist member states in developing national APLs and other relevant policies [14]. This model places people at the center, recognizing the need to understand the population’s AT needs and identify appropriate AP to support participation and integration into society [15]. To implement the APL effectively and have the maximum possible impact, a complete and sustainable provision system, including manufacturing, procurement, distribution, and provision, is needed to ensure availability and affordability (REF). Effective Policies and governance are required to ensure the provision system operates sustainably during these processes [16]. Furthermore, adequate skill-mixed personnel is essential to cater to and guide the service provision at all levels [17]. Therefore, these five interconnected components in an AT system play an important role in APL development [14].

APL development is determined by multiple factors and consequently varies by country. Not only is the number and type of AP needed to achieve population coverage different between countries [18], but also the provision of AT and related services differs substantially by country, particularly in regions and countries where the AT system is fragmented, as has been documented in Africa [19]. For instance, AP provision in different countries may rely on a range of public, private, and charitable organizations [20,21]. Furthermore, while policy frameworks aimed at promoting the rights of PWD exist in all member states, there is variation in their practical implementation to promote AT usage [4].

Research regarding APL development in some low-income countries has been conducted [18,19,20]. However, research comparing the differences between national APL development across these countries is lacking. With an understanding that the interlinked 5Ps play a critical role in the development of national APLs, it is therefore rational to consider comparison of the APLs according to this framework.

This research compares national APL development processes in three countries in Africa which have recently developed APLs—Malawi, Liberia, and Sierra Leone. These countries are comparable in a number of ways. For example, all three countries are considered to be low on the Human Development Index [22,23,24]. Furthermore, they share similar characteristics in AT provision, such as poor access to AT [25,26,27] and donor dependence due to a weak health system [28,29,30]. However, each adopted different actions to improve access to AP and develop APL. In Malawi, a participatory action research project was used to develop the APL [31], while a donor-funded systems strengthening initiative was used in Sierra Leone and Liberia to develop the APL and related policies.

Therefore, the objective of this research is to compare the development of the national APLs in these three countries and to specifically understand how the 5Ps model influenced national APL development across Malawi, Liberia, and Sierra Leone.

## 2. Methods

In this qualitative study, semi-structured interviews were conducted in Malawi, Liberia, and Sierra Leone, allowing respondents to provide in-depth and meaningful insights on APL development [32].

### 2.1. Participants

The participants were selected through non-random purposive sampling and contacted via email. We used existing research networks in these three countries to disseminate information about this research and invited stakeholders to volunteer their participation. Inclusion criteria for semi-structured interviews included involvement in the process of APL development. Participants were included who were representatives of government agencies, disabled people organizations (DPOs), local universities and rehabilitation centers, and other organizations involved in APL development. Participants must have been engaged in the process of APL development in a leadership capacity, be adults (over age 18), and speak English well enough to engage in conversation about the APL development process.

To achieve data sufficiency [33], four participants per country were recruited to represent a range of experiences in the APL development process. The multiple perspectives of different participants from different organizations provided a comprehensive insight into the overall local APL development and strengthened the external validity. Informed consent was obtained from all participants.

### 2.2. Data Collection

A semi-structured interview guide was developed by the research team based on previous work in the area and specifically written to align with the 5P model to ensure all relevant content was covered. The interview guide was piloted and refined prior to data collection. Examples of interview questions can be seen in Table 1. The interviews were conducted online via Zoom or Teams due to limitations associated with travel during the COVID-19 pandemic. The interview duration was between 30 and 90 min. All interviews were conducted in English, recorded with the permission of the interviewees, and transcribed verbatim.

### 2.3. Data Analysis

We used directed content analysis to conduct the analysis [34]. Firstly, a codebook was developed by identifying operational definitions of the five key themes of APL development in the 5Ps model. All transcripts were reviewed carefully and coded using the predetermined categories wherever possible in Atlas.t (v.23). Text that could not be coded into these categories was coded with a new label. Finally, the data from the same and different countries were compared and reported.

### 2.4. Ethical Issues/Statement

Prior to recruitment and data collection, ethical approval was obtained for this research from the Maynooth University Social Research Ethics Committee (2471117), the University of Malawi Research Ethics Committee (P.01/20/10), the University of Liberia Pacific Institute for Research and Evaluation Institutional Review Board (FWA00004853), and the Sierra Leone Ethics and Scientific Review Committee (No number provided). This study was conducted in accordance with the Declaration of Helsinki, and informed consent was obtained from all participants prior to participant interviews.

## 3. Results

A total of 12 individuals participated in the research, representing a range of stakeholder groups across the three countries. Table 2 provides details of the 12 participants, including their gender, country of work, and type of organization they represented in the process of APL development.

This section will demonstrate how the 5Ps identified by the WHO influenced APL development across three countries. We have integrated direct quotes from study participants to demonstrate engagement with each of the 5Ps in the APL development process. We have also provided a summary of the findings across all 5Ps and three countries for ease of comparison in Table 3.

### 3.1. Rationale for APL Development

Each of the three countries introduced a process of APL development to improve access to AT for people in need and provide some guidance for the AT provision. In Liberia, it was noted that “there was currently no national APL for the country, and therefore, there was no guidance as to how to prioritize any sort of resources for procurement or donation of products (Interviewee 5)”. This sentiment was shared by a participant from Sierra Leone, who identified the need for guidance for donations of APs, noting that “AT is not common and relies heavily on donors who provide AT randomly without having to do prior assessment (Interviewee 12)”. One participant (Interviewee 1) from Malawi also recognized that “the needs are diverse, and the government cannot provide everything”, which highlighted the need for a national APL in order to establish those products that should be provided to citizens at a basic level.

### 3.2. People

The people theme highlights that people who use assistive products should be at the center of policy engagement and development. This section included the role of people in three key subthemes: stakeholder engagement, the need for data, and raising awareness. Since the end-users of AP are mostly PWD, all countries identified the engagement of PWD and other relevant stakeholders as important. Malawi began the APL development process by figuring out people’s needs by reviewing the literature and existing data (2018 Census, WHO AT Capacity Assessment, etc.) and reaching out to PWD and their caregivers or family members in the communities. Engagement of caregivers was considered important, as one participant noted that during consultations, they included “people with speaking difficulties, and they would bring their parents, and then their parents would interpret using sign language”, which provided opportunities for meaningful engagement of all stakeholders. Further, to comprehensively understand the challenges faced by PWD, AT service providers and people from government agencies were also interviewed. Liberia placed emphasis on PWD participation and ensured organizations for PWD participated in the technical working group (TWG), each consultation meeting, and break-out groups. The importance of this was highlighted by a participant who noted, “There is nothing for us without us… one of the keys to developing this APL was the inclusion of PWD (Interviewee 9)”, and echoed by a participant in Sierra Leone who also quoted “nothing for us without us (Interviewee 12)”.

The theme of people also includes the need for data on the use and met and unmet needs for assistive products. In some cases, the process of developing an APL included references to sources like the previous population and housing census and the WHO rapid Assistive Technology Assessment (rATA). In Sierra Leone, however, there was a lack of data on disability. One participant noted that it was difficult to know how to address the need for assistive products because “when it comes to disability issues in Sierra Leone, basically it was difficult for us to … have [enough] data (Interviewee 10)”. Therefore, Sierra Leone established a platform that worked closely with DPOs to identify those highly in need of AP. PWD and professionals attended the expert panel and consultation meetings. This process was the first time that disability data had been collected in the country.

Awareness-raising is a final subtheme of the people theme and was demonstrated to be an important factor in effective APL implementation in all three countries. Stakeholders identified awareness of what kind of AP and services are available as a key need. One stakeholder in Malawi noted that “we need a lot of awareness to involve more the public in terms of bringing awareness to the communities on the need for support [and] the provision of assistive devices (Interviewee 4)”. It was also felt to be important to raise awareness during the process of APL development “because people need to know what the services are what are the benefits they should get from the APL (Interviewee 12)”. Despite all three countries’ efforts to involve as many as possible to ensure no group is left behind, stakeholders acknowledged some issues may have been missed. For instance, it was noted by respondents that stakeholders who were engaged in Liberia were mostly from urban areas that could not comprehensively represent AT users in rural areas.

### 3.3. Personnel

Human resources were regarded by stakeholders as one of the most critical factors in developing and implementing APL and making the whole provision system function well. Two subthemes were discussed with respect to personnel for assistive technology provision: personnel training and personnel coordination.

In Malawi’s Disability Policy and Medical Rehabilitation Policy, personnel training was identified as one of the key issues and strategies. Malawi lacked trained personnel at all levels of the healthcare system. Therefore, the Ministry of Health had started training personnel at home and abroad. However, issues regarding personnel training emerged in Malawi without a budget, proper guidance, and lack of coordination. Given the lack of appropriately trained personnel, stakeholders noted that it would be important to provide training to all individuals who interact with an assistive technology user, noting “the family or guardians who assist the person with disabilities should be trained as well (Interviewee 2)”.

Liberia also identified a significant gap in personnel and outlined several interventions regarding personnel training in their AT roadmap. These interventions included “scaling up the training of health workers and also social workers … developing both pre-service and in-service training curricula, [and] developing service delivery standards… (Interviewee 5)”. With these interventions, Liberia intended to use them to mobilize and make maximum use of available resources to carry out the training. Furthermore, it was clear that the development of a list alone was not sufficient to effect change. Interviewee 5 also noted that it would be important to “strengthen capacity of local institutions to conduct training…” and that this should be “based on this priority list to ensure that we’re focusing AT training on priority products that are on this current list so that we make maximum use of the resources that we have … instead of sending students abroad”. People are currently being trained to provide AT in a pilot project using the WHO Training in Assistive Products Program (TAP). Furthermore, there were numerous organizations working in the AT field with little coordination for provision.

Sierra Leone was developing a roadmap that served as a guide for training personnel. They also created a TWG involving different stakeholders within the MoHS. Respondents hoped to train people in different areas to produce AP locally and provide services. They suggested stakeholders complement each other to avoid duplicating AT services. Additionally, they were cooperating with serval organizations working on specific assistive products so these organizations could provide some training for the local teams. This was important as it was noted that increasing production capacity was not sufficient, as this “capacity cannot go without the human resources (Interviewee 12)”. Understanding how these roles overlap and complement one another highlights the need for the second subtheme—personnel coordination.

All three countries identified a lack of coordination between AT providers as challenging and an issue that would need to be addressed for the effective implementation of an APL. It was often the case that some AT services were provided by NGOs and others by government agencies, as in Malawi, where Interviewee 4 noted, “We have NGOs doing their own things, and the ministry doing its own things now”. One interviewee (9) in Liberia noted that “if the coordination was … strong in the first place, they would have all the different groups [who] provide different categories of AT and services based on their own interests”. A participant from Sierra Leone felt that “a kind of platform where organizations come and play a key role in that area, which is to complement the effort of the government (Interviewee 12)” could be one approach to addressing the lack of coordination between personnel and improving access to services once an APL was in place.

### 3.4. Products

The number and the type of AP among each of the three countries’ APLs were different. There were 23, 33, and 70 APs in Malawi, Liberia, and Sierra Leone’s APL, respectively. Three subthemes were evident when discussing products: the standards by which products were chosen, procurement sources, and regulation of product quality.

Different standards by which products were chosen contributed to differences between the three APLs. In Malawi, researchers from the Center of Social Research and Maynooth University conducted research in six districts to understand actual AP needs and other AP-related information in Malawi’s setting. Then, an action research group composed of stakeholders from the Center of Social Research, the Ministry of Health, DPOs, and international and local organizations discussed what AP should be prioritized considering affordability, availability, accessibility, and geographical context. It was clear to this group of stakeholders that “assistive devices suitable for the terrain in the northern regions may not be suitable for other environments, [so] geographical context is taken into account (Interviewee 3)”. The consensus was that the APs should be user-friendly and satisfy people’s needs; therefore, proper assessment for users is needed. Furthermore, the capacity of human resources was also considered. However, due to financial constraints, the government was not able to afford all the needed AP and human resources. Therefore, they “made a balance to ensure the majority of people’s needs and the capacity [of the AT system] and decided at least to provide the very basics (Interviewee 2)”; this resulted in the choice of 23 products representing the basic needs needed AP, which could be addressed within the country’s current capacity.

In Liberia, a TWG consisting of AT users, providers, and stakeholders prioritized products based on the local context. Then, according to the five prioritization criteria and their weights, including impact on quality of life, total need, operational feasibility, cost, and unmet need, 33 APs with the highest scores were approved to be on the list. Among the five prioritized criteria, cost (economic conditions of users as well as training costs) and operational feasibility (geographic factors as well as the capacity of health and social welfare systems) were the main factors. Operational feasibility was considered “not just from a user perspective but from the system perspective (Interviewee 6)”, as well.

Sierra Leone held three consultative meetings and one final validation meeting and established a platform for priority AP selection. An expert panel group was created comprising AT experts, users, and stakeholders from different regions. Consequently, 70 out of 308 products were determined using a qualification framework with four criteria: disability conditions, role in daily function from the user’s perspective, affordability, and capacity analysis. Population, geographic details, and available human and material resources were also analyzed.

When discussing the source of assistive products, all three countries noted they rely heavily on donors, and most AP are imported, causing challenges in the procurement process. One interviewee in Sierra Leone (10) noted that “they get the devices from donors that they do not have a say in … because economically they cannot purchase devices”. In Malawi, it was noted that “most APs, especially for eye care, hearing aids, self-care, and education, they are imported (Interviewee 4)”. This was also true for Liberia, where “the majority of ATs, whether it is in the public sector or the private sector, are donated by partners and imported (Interviewee 5)”.

Donated products often led to challenges with product quality, the third subtheme. In Malawi, the government has been asked to put in a budget to scale up local production in an effort to address this concern. However, quality regulations are lacking except for some professional guidance, and discussions on quality standards are ongoing within the Malawi Bureau of Standards, but “it has not been finalized yet” (Interviewee 2). While participants from Liberia noted they have the capacity to manufacture AT locally, they lack the raw materials. Liberia Medical Health Regulatory Authority (LMHRA) and Liberia Medical and Dental Council are the current overall regulatory authorities for product quality. However, quality standards for AP on the APL still need to be developed. It was specifically noted that “there’s a need to develop technical specifications and quality checklists for the procurement and importing or donation of products with a focus on the APL (Interviewee 5)”. Few APs were manufactured in Sierra Leone, and they had a challenge in the supply chain. UNICEF played a role in the procurement process and is currently developing a list of drug and medical supply products. Sierra Leone was trying to incorporate the APL into the list so that the government could conduct the procurement. Furthermore, Sierra Leone viewed product quality as critical, particularly because of environmental conditions. One interviewee noted that “we have poor roadwork; for persons with a disability using wheelchairs, some of the areas are not accessible, so the quality of some of these products that they use is very key (Interviewee 12)”, and highlighted the importance of developing quality guidelines for APL.

### 3.5. Provision

The theme of provision centers on the health and social care systems where AT is provided. This theme focuses on three subthemes: AT systems and the organizations who are involved in them, challenges in AT provision processes, and AT services provided for products on the APL.

There are a variety of *AT systems* engaging a range of organizations that provide AT. For example, Malawi has three levels of government healthcare systems that provide AT: rural health centers, district hospitals, and the central hospital. Specifically, the Ministry of Health, Ministry of Gender, Children and Community Work and Social Welfare, and Ministry of Education are responsible for AT provision. Malawi Against Physical Disabilities, an NGO, manufactures and distributes AP outside of government channels. These, combined with provision by international NGOs and donors, have led to provision systems that are poorly coordinated (as discussed in the section on personnel). In Liberia, “AT provision occurs in both the public and private sectors, with very few facilities currently providing AT (Interviewee 6)”. In the public sector, the key service delivery points are the Monrovia Rehabilitation Center, John F. Kennedy Medical Center (JFKMC), two larger rehabilitation facilities outside of Monrovia, and a large eye center. Among them, JFKMC serves as a referral center. Additionally, some international organizations, local organizations like LMHRA, and private individuals or facilities are involved in the provision. The APL will be integrated into the LMHRA documentation, allowing personnel and the government to quantify, procure, and distribute AP. Furthermore, Liberia intends to integrate data and information regarding AT and services into the existing health system for better provision. Sierra Leone lacks a well-established provision system and is therefore developing policies to support the provisioning process; however, it notes that all relevant “organizations are working closely with the Ministry of Health (Interviewee 12)”, suggesting there is potential for collaboration and a coordinated effort.

All countries shared some common *challenges regarding provision*. Coordination is a challenge, and in some cases, such as Sierra Leone, significant budget waste and duplication have been reported. Stakeholders from all countries reported a lack of budget and human resources to procure needed AP and provide services. One participant from Malawi (4) noted that at the moment there is “no budget line [for AT], so then you cannot be assured that the assistive technology will be there all the time”. There were also country-specific challenges. In Malawi, there are no regulatory mechanisms or guidelines for service provision, resulting in some APs not meeting people’s needs. For example, as described by one participant in Malawi, there are no “developed regulatory mechanisms yet … sometimes people just give wheelchairs and give crutches without looking at the specific condition of the individual (Interviewee 4)”. Further difficulties exist as demand exceeds supply and technical capacity is limited. The provision system in Liberia was fragmented and vulnerable to impact from external factors, as has been experienced in the COVID pandemic. One participant described how the number of service delivery points was insufficient, noting “there are enormous gaps between AT service delivery points and the population that requires AT (Interviewee 6)”. Hence, a referral system has been suggested to be developed to better link providers and users in both the private and public sectors. While recently “there are more services being provided … in terms of the scope of service or the package of service, it is really not standardized across the various private facilities (Interviewee 5)”. In Sierra Leone, the supply chain was unstable; therefore, the availability of AP cannot be guaranteed, even if the services are available, so there is an emphasis on being ready to “really scale up production and to ensure there is the availability of abundant products that are needed in the country (Interviewee 12)”.

*AT services* include assessment, supply, fitting, training, repair and maintenance, and follow-up. In Malawi, the “Disability Policy and Medical Rehabilitation Policy prioritize those services: assessment, supply, fitting, training (Interviewee 4)”. However, they reported inadequate personnel to do the assessment. Therefore, training more personnel with the necessary skills and knowledge to provide services is required. There was also an identified need to “periodically carry out satisfaction surveys, just to ensure that people with disabilities are happy with the quality and range of products available on the market (Interviewee 2)”. Aligned with this, stakeholders in Liberia reported a patient satisfaction survey informing their service delivery in 2013. However, the frequency of these surveys is limited by funding. To address service needs, the AT roadmap in Liberia included training more skilled personnel and ensuring additional facilities for AT services. These “recommendations from the ministry are to increase the AT workforce or increase the number of AT specialists who are able to conduct repair and maintenance and to ensure that additional facilities are included where these services can be provided to clients (Interviewee 5)”. A clear guideline also indicated the need for service provision at the community level. To address this gap, the “Medical Rehabilitation Center in Liberia will train personnel in different health facilities to distribute, to fit, to assess, and deliver (Interviewee 6)”. In Sierra Leone, personnel able to produce, assess, and prescribe were in short supply. Ideally, providers will work with the National Rehabilitation Center under the MoHS to provide AT services. There are currently plans to establish repair centers that can help users train, assess, and repair. Stakeholders also expressed a wish to have a digital data system for tracking users’ information and following up.

### 3.6. Policy

Malawi has a national Disability Mainstreaming Strategy, which was developed in 2017, but does not have a separate policy on AT. Hence, the APPLICABLE project “the aim was to develop a policy on assistive technology (Interviewee 3)” and ensured research was used to guide the APL development process. After consulting with the Ministry of Gender, Malawi decided to include AT as part of the Disability Policy rather than developing a standalone AT policy. Hence, they were reviewing the draft Disability Policy, which included AT and APL as key priority areas. At the time of this research, it was awaiting approval by the government. Additionally, there was a medical rehabilitation policy where AT services are included.

In Liberia, the APL was integrated into the essential package of health services to facilitate the availability and delivery of AP on the list. Moreover, Liberia created an AT roadmap outlining activities and interventions to improve the provision process and scale up AT access, including establishing referral systems, encouraging better coordination among sectors, and developing guidelines and service delivery standards to standardize the AT provision. While “the AT roadmap itself is not really a policy, (Interviewee 5)” it does present a clear implementation plan to increase access to AT. Due to limited resources, not all of the AT roadmap recommendations are currently being implemented. Participants noted their hope the APL and AT roadmap will be actualized and implemented. Furthermore, the APL will align with the key policy objective of the National Action Plan for PWD, which emphasizes increasing access to AT. Because policy development is an ongoing process, they will develop relevant policies and integrate the APL into other significant policies, such as the LMHRA guidelines and quantification guidelines for the ministries.

In Sierra Leone, there is no existing policy on AT. However, the projects there were developing the national AT policy and the APL simultaneously. In this way, “the documents are not isolated; they speak closely to each other (Interviewee 12)”. They attempted to link everything together when developing policies so that every policy is introduced into the mainstream health system. Although the APL is completed, there are outstanding tasks, such as drawing a roadmap as guidelines and developing national procurement guidelines. These “national procurement guidelines … are very crucial because [they] would define the standards for the different products we have on the APL list (Interviewee 11)”. While each of the three countries is in different circumstances regarding AT policy, it was clear the government and civil society were engaged in attempting to improve the situation.

### 3.7. Actions to Implement the APL

Regarding actions to implement APL, stakeholders from all three countries suggested similar strategies. First, they highlighted the need for a broad range of stakeholders to cooperate. Second, more personnel with the necessary skills and knowledge should be trained according to the local personnel landscape. Third, they need to maximize the benefits from limited resources focused on the priority AP on the list. Moreover, Malawi suggested the first action is “to finalize the policy (Interviewee 4)”, and then “to create awareness amongst the person, the users, so that they know what is available on the market and how they can access the product (Interviewee 2)”. In Liberia, there was an identified “need to develop training materials and service standards for provided … and to develop technical specifications and quality checklists for the procurement and importation or donation of products with a focus on the products on the APL (Interviewee 5)”. Finally, participants from Sierra Leone emphasized the importance of developing the national procurement guidelines and proposed mainstreaming the APL. Mainstreaming was considered particularly important “because if it is left in isolation, then it will not be definitely properly implemented (Interviewee 10).

## 4. Discussion

This study aimed to compare and analyze the differences between APL development across Malawi, Liberia, and Sierra Leone. We looked into the development processes and how the five components in the 5p model apply to it. We found that APL development is a complex and continuous process that integrates information related to each of five interlinked aspects: people, policy, personnel, product, and provision.

APL development comprises two stages: APL formation and implementation. People significantly influence formation differences, while personnel are crucial for implementation. Finally, countries develop effective policies to guarantee a sustainable provision system.

AT users, in this case, particularly people with disabilities, directly shape differences in APL formation. In this study, we found that across all three countries, product selection criteria are shaped through joint stakeholder consultation and available data sources. Stakeholders’ involvement in understanding people’s needs was a common characteristic across all three countries, although methods varied. The lack of standardized data collection in LMICs potentially leads to this variation [7]. Beyond needs, contextual factors and costs impact the actual number of products in APL. We found substantial differences in the number of products across the three countries. Contextual factors and costs impact the number of products in APL [35], with funding challenges affecting AT availability [1]. Cost considerations were also found in all three countries, consistent with the literature, which suggests costs also influence access to assistive products [36], particularly for products outside APL and for people in settings with limited social welfare support. Furthermore, we found that a lack of awareness about the importance of AT persists at all levels, including policymakers, caregivers, and potential beneficiaries, consistent with findings from the literature [37], necessitating efforts to enhance a broader understanding of AT benefits and promote stakeholder participation in mainstreaming APL and its implementation.

Our findings suggest that personnel were regarded as an important factor in APL implementation, particularly in training and collaboration. Personnel training is multidisciplinary, with many personnel being trained in various AT areas [38]. Consistent with our findings, the literature suggests that lower-resourced countries may lack trained AT specialists and rehabilitation practitioners, and those who provide AT services may be untrained [17], resulting in service delivery disparities, AP abandonment, and unintended consequences [39,40]. Additionally, we found that contextually relevant training programs are needed for diverse personnel and groups, considering the characteristics of AP and user disabilities. However, research suggests that funding constraints often hinder training program delivery [41]. Moreover, we found significant complexity and lack of coordination across AT provision systems, primarily related to the fact that AT is provided across serval sectors. In related research, Smith et al. identified limited cooperation between different service organizations, and they proposed that cooperation between organizations should be promoted to avoid the unnecessary overlapped roles of each organization and facilitate the specialized skill sets of personnel [17]. Our research similarly identified government leadership as crucial for coordinating collaboration among organizations.

Personnel drive APL implementation, while policies provide direction. We found a lack of AT-specific policies in Malawi, Liberia, and Sierra Leone, each of which is at a different stage of policy development. Developing sustainable AT policies is complex, requiring a cross-sectoral, people-centered systems mindset [12]. To achieve that end, we found that Liberia has developed an AT roadmap outlining many interventions and activities in six thematic areas to clarify the roles of various organizations within them and the related resource mobilization. Similarly, Sierra Leone is developing a roadmap to achieve policy and programmatic outcomes. Our research identified a slightly different approach in Malawi, where the mainstreaming approach places assistive technology in existing relevant policies. This is consistent with MacLachlan et al.’s suggestion that AT policies should be linked to other persuasive policies. Comprehensive policies encompassing personnel training, procurement guidelines, product and service standards, and provision evaluation are imperative for mainstreaming and implementing APL, ensuring a sustainable provision system [2].

According to our findings, current sources of assistive products primarily include external procurement, local production, and donations. However, we also found that the process of sourcing these products is not guided by relevant policies, standards, or specifications. Developing procurement guidelines, product standards, and technical specifications can guide production, procurement, and donations [42]. Policies are essential for standardizing training, competency, and knowledge objectives for personnel involved in AT services. Qualified products and standardized AT services, coupled with clear departmental roles and cooperation, facilitate a robust provision system. Furthermore, data play a crucial role throughout APL development, influencing decision-making and policy formulation [2,43]. Our research suggests that limitations in available data can challenge the process of APL development, particularly where limited data on disability and assistive technology use persists. Research and data help in estimating demand, optimizing processes, and avoiding duplication through a data-sharing system. Ebuenyi et al. (2021) recommended the development of mechanisms that will improve the use of existing data on AT in all countries for the efficient and effective development of AT ecosystems [43].

In summary, our research found that in all three countries, people’s needs determined AP selection on the APL, prioritizing limited resources for affordable priority products. Skilled personnel and policy are crucial for a sustainable provision system, with personnel ensuring quality service and policies guiding APL implementation. Additional contextual factors, including existing policies and systems, account for differences in approach and differences in the resulting APLs. Thus, the interactions between the 5Ps, in combination with different national contexts, have resulted in varying APL developments.

### 4.1. Limitations

Two main limitations can be outlined. Firstly, due to the small sample size of this study, the richness of data may not be achieved, causing an incomplete understanding of APL development in the three countries. However, according to Guest, Bunce, and Johnson [44], data sufficiency typically occurs with six to twelve in-depth interviews, a condition met in this research with twelve semi-structured interviews. Furthermore, all interviews were conducted online, potentially impacting quality, with technical issues such as internet connectivity problems causing interruptions and disrupting the flow.

### 4.2. Recommendations for APL Development

This study suggests attention to specific areas in APL development.

- Identify and prioritize the needs of the population by evaluating existing official data or adopting the rATA developed by the WHO [43].
- Ensure a sustainable provision system, including trained personnel in different AT areas, guidelines or strategies in effective policies [45], and connections between relevant AT policies.
- Mobilize limited resources to focus on priority products to ensure the developed APL, resources, and efforts are focused and coordinated [7].
- Establish a public information platform to improve public information transparency, thereby improving service efficiency.

Review and update the APL at intervals based on actual conditions since developing the APL is a continuous process.

## 5. Conclusions

The five components of the 5Ps model have varying effects on APL development and subsequent implementation. The component of people directly influences the differences in APL formation in three countries. The other four components affect how the three countries have taken actions to implement the APL. Policy is the overarching factor in the other three components. National context also affects APL differences. Despite sample limitations, this study provides valuable insights into how the 5Ps model influences APL development disparities. As this is the first study to compare the differences in APL development, these findings may help to advance APL development in these three countries and others.

## Figures and Tables

**Table 1:** Example of interview questions.

Interview Questions
What were the most important factors that you considered in the APL development [economic factors/demographic factors/healthcare system]?
Could you describe how you ensured that AT users were engaged in the APL development?
In the APL development, did you have access to the data regarding AP needs in … [Country’s name] and how did you use the data?
We noticed that [name of country] has … products on the APL, and these are not necessarily the same as those on the WHO list. Could you explain how you determined which products should be included and why these might differ from the global list?
What kind of actions should be taken to implement the APL?

AT: Assistive Technology; APL: Assistive Product List; AP: Assistive Products; WHO: World Health Organization.

**Table 2:** Demographics of interviewees.

Interviewees	Organization	Gender	Country
Interviewee 1	Government Ministry	Female	Malawi
Interviewee 2	Local NGO	Male	
Interviewee 3	University	Female	
Interviewee 4	Independent Consultant	Male	
Interviewee 5	International NGO	Female	Liberia
Interviewee 6	Health Centre	Male	
Interviewee 7	Health Centre	Male	
Interviewee 8	Health Centre	Male	
Interviewee 9	WHO	Male	
Interviewee 10	International NGO	Male	Sierra Leone
Interviewee 11	International NGO	Male	
Interviewee 12	Health Centre	Male	

NGO: Non-Governmental Organization.

**Table 3:** Differences in APL development across Malawi, Liberia, and Sierra Leone.

Parameters	Malawi	Liberia	Sierra Leone
**People**			
Stakeholders	DPOs, Ministries, Policymakers, Universities	DPOs, Ministries, Policymakers, CHAI	DPOs, Ministries, Policymakers, CHAI
Use of research	(1) The survey conducted by the Center for Social Research and APPLICABLE Research, (2) rATA, (3) Country situation analysis conducted by CHAI, (4) 2018 population and housing census	(1) Population and housing census, (2) PWD needs assessment of 2009, (3) Labor workforce surveys, (4) Country situation analysis conducted by CHAI, (5) rATA	Yes, but not clear
**Products**			
Number	23	33	70
Sources	Some manufacture locally, some imported, and out-of-pocket spending	Most are donated by partners and imported	Most from external donors
Criteria for Selection	(1) Geography, (2) Provision capacity, (3) Affordability, (4) Accessible	(1) Impact on quality of life, (2) Total need, (3) Operational feasibility, (4) Cost, (5) Unmet need	(1) Disability conditions, (2) Role in daily function from the user’s perspective, (3) Affordability, (4) Capacity analysis
**Personnel**			
Skilled personnel	Shortage	Shortage	Shortage
Coordination	Exists but weak	Based on interests	Ministries worked closely with organizations in a platform
**Provision**			
Challenge	(1) Coordination, (2) Budgetary constraints, (3) Quality control, (4) Human resources, (5) Supply chain	(1) Coordination, (2) No standard for products and services, (3) Insufficient service delivery points, (4) Budgetary constraints, (5) Human resources	(1) Coordination, (2) Supply chain, (3) No guideline for procurement and product standards, (4) Lack of AT services, (5) Budgetary constraints
AT services	Inadequate, especially in assessment and repair	Inadequate, especially in repair and maintenance	Inadequate, especially in production and repair
**Policy**			
	Disability policy (Awaiting approval), Medical rehabilitation policy	AT roadmap (Some interventions have been put into practice)	AT roadmap (Developing)

DPO: Disabled Peoples’ Organization; CHAI: Clinton Health Access Initiative; APPLICABLE: Research Project in Malawi; rATA: WHO rapid Assistive Technology Assessment; PWD: Person with Disability; AT: Assistive Technology.

## Data Availability

The raw data supporting the conclusions of this article will be made available by the authors on request.
